# Brief Interventions Implementation on Alcohol from the European Health Systems Perspective

**DOI:** 10.3389/fpsyt.2014.00161

**Published:** 2014-11-11

**Authors:** Joan Colom, Emanuele Scafato, Lidia Segura, Claudia Gandin, Pierluigi Struzzo

**Affiliations:** ^1^Program on Substance Abuse, Public Health Agency of Catalonia, Barcelona, Spain; ^2^Istituto Superiore di Sanità, Rome, Italy; ^3^Regional Centre for the training in Primary Care (Ceformed), Monfalcone, Italy; ^4^Department of Life Sciences, University of Trieste, Trieste, Italy

**Keywords:** alcohol, brief interventions, health system, empowerment, resilience

## Abstract

Alcohol-related health problems are important public health issues and alcohol remains one of the leading risk factors of chronic health conditions. In addition, only a small proportion of those who need treatment access it, with figures ranging from 1 in 25 to 1 in 7. In this context, screening and brief interventions (SBI) have proven to be effective in reducing alcohol consumption and alcohol-related problems in primary health care (PHC) and are very cost effective, or even cost-saving, in PHC. Even if the widespread implementation of SBI has been prioritized and encouraged by the World Health Organization, in the global alcohol strategy, the evidence on long term and population-level effects is still weak. This review study will summarize the SBI programs implemented by six European countries with different socio-economic contexts. Similar components at health professional level but differences at organizational level, especially on the measures to support clinical practice, incentives, and monitoring systems developed were adopted. In Italy, cost-effectiveness analyses and Internet trials shed new light on limits and facilitators of renewed, evidence-based approaches to better deal with brief intervention in PHC. The majority of the efforts were aimed at overcoming individual barriers and promoting health professionals’ involvement. The population screened has been in general too low to be able to detect any population-level effect, with a negative impact on the acceptability of the program to all stakeholders. This paper will present a different point of view based on a strategic broadening of the implemented actions to real inter-sectoriality and a wider holistic approach. Effective alcohol policies should strive for quality provision of health services and the empowerment of the individuals in a health system approach.

## Introduction and Methods

This is a review study to discuss how screening and brief interventions (SBI) for harmful alcohol use and alcohol dependence can be better embedded in health system (HSys) and implemented effectively. To do so, first the challenges for AUD treatment and the HS responses, as recommended by World Health Organization (WHO), are presented, followed by a review of the existing SBI evidence and the cases of SBI wide implementation and finally some future directions toward the achievement of this objective are proposed. Even if there is still considerable confusion in the literature regarding the SBI evidence, SBI programs have been implemented nation-wide in some countries with some positive results and could be seen as important cornerstones to implement more broaden national policies on alcohol risk reduction. There is, however, a long road ahead. The situation regarding alcohol use is changing dramatically, with the frequent presence of binge drinking in youths, the constant of poly abuse, typically of novel psychoactive substances (1), and the regular report of co-morbid psychiatric disorders (2). The results of the existing experiences urge HSys to move beyond a focus on individual (professional’s and patient’s) behavior toward implementing policies having into account a wide range of social and environmental interventions, the so-called health promotion, as defined by the WHO^1^.

The novelty that this review will bring to the reader mainly refers to a point of view that focuses on “what works” and also on broadening future actions with real inter-sectorial strategies encompassing health services (HS) with other sectors of the society and addressing the individual alcohol user with SBI included into a broader, holistic, risk-reduction approach. As alcohol is a complex issue, the general idea is to move from a health service-centered to a broader HSys intervention.

## Health Services in Respect to Brief Interventions for Alcohol Use Disorders in Europe: The Challenge of the Treatment Gap

According to the WHO, HS, the most visible functions of any HSys, include all services dealing with the diagnosis and treatment of disease or the promotion, maintenance, and restoration of health. In this sense, WHO have stressed that HS for AUD have the following objectives:

- provide prevention and treatment interventions to individuals and families at risk of, or affected by, AUDs and associated conditions;
- inform societies about the public health and social consequences of hazardous and harmful alcohol consumption (HHAC);
- support communities in their efforts to reduce HHAC;
- advocate effective societal responses.

Despite the efforts made by WHO and all the countries to improve AUD treatment, evidence still shows that the so-called treatment gap is huge. From one side harmful alcohol users are still socially stigmatized and do not seek treatment and from another access to effective alcohol treatment services is limited in many European countries. It has been estimated that only 1 in 20 of those with HHAC are actually identified and offered brief advice by a primary care service provider. Similarly, <1 in 20 with a diagnosis of alcohol dependence has actually seen a specialist for treatment (3).

Taking into account this reality and the ambitious AUD treatment objectives, it is clear that a cultural change in the way alcohol problems are seen is needed. As a consequence of that we need to mobilize and involve of a broad range of players inside and outside the health sector, sufficiently strengthened and properly funded in a way that is commensurate with the magnitude of the public health problems caused by HHAC. This means broadening the horizon to a much wider HSys approach^2^(3).

The provision of early intervention and treatment services is a key part of any comprehensive policy framework to reduce alcohol harm (4). The WHO “Global Strategy to Reduce the Harmful Use of Alcohol,” 2010, lists National HSys’ response as one of its key priority policy areas (5): (1) leadership, awareness, and commitment; (2) HS’s response; (3) community action; (4) drink–driving policies and countermeasures; (5) availability of alcohol; (6) marketing of alcoholic beverages; (7) pricing policies; (8) reducing the negative consequences of drinking and alcohol intoxication; (9) reducing the public health impact of illicit alcohol and informally produced alcohol; and (10) monitoring and surveillance.

The portfolio of policy options and interventions recommended by the WHO for HSys’s response area include

(a) increasing capacity of health and social welfare systems to deliver prevention, treatment and care for AUDs, including support and treatment for affected families, and support for mutual help or self-help activities and programs;
(b) supporting initiatives for SBI for HHAC at primary health care (PHC) and other settings including initiatives among pregnant women and women of child-bearing age;
(c) improving capacity for prevention of, identification of, and interventions for individuals and families living with fetal alcohol syndrome and a spectrum of associated disorders;
(d) development and effective coordination of integrated and/or linked prevention, treatment, and care strategies and services for AUDs, including drug-use disorders, depression, suicides, HIV/AIDS, and tuberculosis;
(e) securing universal access to health, enhancing availability, accessibility, and affordability of treatment services for groups of low socio-economic status;
(f) establishing and maintaining a system of registration and monitoring of alcohol-attributable morbidity and mortality, reported on a regular basis;
(g) provision of culturally sensitive health and social services as appropriate (5).

In respect to SBI for alcohol-related problems, HS are central to tackling harm at individual level among those with AUDs and other conditions caused by HHAC. The outcome expected by the WHO action plan to reduce HHAC 2012–2020 is a progressive reduction in the gap between the number of people who would benefit from alcohol consumption advice to reduce or prevent harm, engagement in social rehabilitation programs or treatment for AUDs and the number who actually receive such advice or treatment to be monitored (using as indicators the proportion of the adult population with HHAC, and of the population with HHAC who have received therapy and advice from a primary care provider to reduce their alcohol consumption) (3).

The health sector and the social welfare, education, and workplace sectors have real opportunities to reap both health gain and financial savings through the widespread implementation of SBI programs that have been shown to reduce ill health and premature death subsequent to HHAC and the implementation of evidence-based treatment programs for AUDs (3).

It is estimated that of the total cost to the NHS from alcohol harm each year, only around 2% is spent on identifying and treating AUDs. Implementing SBI does not require extensive training and can be delivered in a variety of settings: emergency and hospital care, PHC, schools, job centers and pharmacies, social services, accident, workplace settings, and prisons (6).

There is a strong evidence to support the benefits of widespread implementation of SBI provided by Primary Care and other health or social care professionals while, for alcohol dependent subjects, access to effective treatment services can play a vital role in both recovery from and management of AUDs (6).

According to the WHO, Governments should support SBI programs and referral to specialist services by ensuring that

- clinical guidelines for such interventions are widely available;
- primary care providers receive the training, clinical materials/tools, and advice they need to set up such programs;
- primary care providers are adequately reimbursed for the interventions.

Furthermore, primary care providers should be encouraged to undertake this intervention when they are supported by specialist services to which they can refer problem drinkers. Thus, specialist services for AUDs should be available and evidence-based non-pharmacological and pharmacological treatments should also be offered to those who have been assessed as likely to benefit. Data from a number of recent European projects show that PHC providers considered resources currently allocated for training and delivery of early intervention and treatment not sufficient. The trend has been to move away from lengthy inpatient treatment toward outpatient and community-based one (3).

The current challenge for HS in Europe is how to stick to the values of universality, access to good quality care, equity, and solidarity taking into account the growing challenges (increased costs, population aging, rise of chronic diseases, and multi-morbidity leading to growing demand for healthcare, shortages, and uneven distribution of health professionals, health inequalities and inequities in access to healthcare) and bearing in mind the economic crises that are putting endanger the HS’s sustainability. EC stresses that HS reforms should focus on (1) strengthen their effectiveness, (2) increase their accessibility, and (3) improve their resilience meaning capable to adapt effectively to changing environments, tackling significant challenges with limited resources.

## Widespread Implementation of SBI Programs: What the Evidence Says

Screening and brief intervention is an effective and cost-effective method for treating subjects with HHAC in PHC. Evidence on the reduction of alcohol consumption is consistent, but its impact on alcohol problems is less clear (7). There are, however, a lot of issues on SBI that need further research: identifying the effective components, their utility among dependent drinkers, assessing fidelity to contents, skills needed to implement SBI, and how professionals may best acquire them. SBI effectiveness in the context of chronic diseases should be tested and demand for alcohol SBI may also be potentiated (8).

Despite its effectiveness and strong research evidence to support its implementation in real-world clinical settings, widespread implementation of SBI has occurred in very few places and it is still unclear if the programs will be sustained. In addition, little is known about the most successful strategies for widespread SBI implementation. Babor et al. (9) found that the effectiveness of different implementation models depends on complex provider and organizational characteristics. Thus, the ability of PHC centers to implement SBI was correlated with prior SBI expertise, centers stability, and number of clinicians trained and negatively correlated with lack of provider time, staff turnover, and competing priorities. Authors suggest that the best option is to combine different methods or multi-faceted strategies (10). In his revision, Williams et al. (11) analyzed under the consolidated framework for implementation research (CFIR) (12) eight implementation programs in nine different countries. He found SBI rates varied a lot and were non-comparable because of the use of different measures, scopes, and durations. He concluded that the use of strategies related to inner setting (“features of the structural, political, and cultural context through which the implementation process proceeds”), outer setting (“economic, political, and social context in which an organization resides”), and process implementation domains could be positively associated with higher screening rates and thus to successful implementation.

So far, institutionalization of SBI, which is sustained and nation-wide extensive SBI activity, has only been reported by programs in Finland, Sweden, and Scotland. Seppänen et al. (13) found an increase over the years and a high percentage of physicians (78.5%) offering BI at least occasionally. Among the factors associated with high BI was long experience in PHC and being a PHC specialist.

Studies in Sweden and Finland have shown that only a minority of the population has been asked about their drinking by PHC professionals and a minority of risky drinkers has been advised to cut down. Nilsen et al. (14, 15) found that only 14–20% of the overall sample who had visited a physician in the last year recalled having received an alcohol enquiry. Reduced alcohol consumption was reported by 12% and especially among those who were exposed to a 1–10 min (versus 1 min) conversation on alcohol. In the case of Finland, only one-third recalled being asked, and 37% had been given advice (16).

In England, Kaner (17) claims that national alcohol strategies alone do not result in a wide-scale SBI activity and for that to happen it is needed to create necessary conditions (shaping the policy and commissioning) in which brief interventions become meaningful for those working in clinical practice. Authors also suggest considering system-level factors that influence drinking behavior and policy-level interventions (minimum price per unit for the alcohol sales, restrictions on the density of outlets, etc.) that can reinforce or complement practitioner-level interventions. It was recognized that SBI activity could not occur in public HSys without the prioritization, the support of senior management, or without appropriate resources, including training and support and the definition of integrated care pathways for alcohol prevention and treatment.

Heather (18) also advised that SBI alone, especially with such low levels of people screened and of risky drinkers advice, would be unlikely to result in public health benefits and recommends proposing opportunistic screening to ensure acceptability of SBI programs and to research population-level effects of SBI, especially in combination with other alcohol control measures.

In Scotland (19), where a specific 3-year target (HEAT H4) on brief intervention (149,449 from April 2008 to March 2011 and 61,081 from April 2011 to March 2012) was established to support population-wide implementation, it was proven to be possible to reach it nationally in all priority settings and healthcare staff saw SBI as a worthwhile activity. The reach and impact of the initiative was mixed across Scotland and gaps in coverage were noted, especially in rural and remote areas in relation to age/gender groups who less frequently attended mainstream services.

According to Angus et al. (20), SBI is highly cost-effective for brief intervention at next registration as well as at next general practitioner consultation. Thus, investments in SBI programs not only improve health and save lives but also save HSys money by two levels of action:

- Offering brief interventions to 60% of the population at risk. This ambitious target would require that every patient who receives primary care services would be offered these interventions, irrespective of the reason for the consultation, and a greater investment in training and supporting primary care providers.
- Offering early brief interventions to 30% of the population at risk of HHAC. It can be achieved by putting into place appropriate systems, including provider training, so that every patient who registers with a new primary care provider, receives a health check, consults a provider about particular disease categories (such as hypertension or tuberculosis) or goes to particular types of clinics is offered these interventions. At this level of action, as alternative to standard face-to-face interventions, web-based approaches and self-help guidance could be considered. In this regard, a number of studies are underway to test the effectiveness and the acceptability of this new approach to know if the provision of facilitated access by primary care providers to an alcohol reduction website could significantly increase brief intervention rates by offering a time-saving alternative to face-to-face intervention. These studies include the randomized controlled trial carried out in the Friuli Venezia Giulia Region, Italy (21).

## Case Studies Presentation: HSys for BI in Six European Countries

A literature search showed that only six countries/regions in Europe have been working on the wide implementation of SBI on alcohol, i.e., they have invested intensive and continuous efforts aimed at institutionalizing that programs and their initiatives have been endorsed by national laws, policies, or guidelines. In other countries, such as Slovenia, Czech Republic information is missing. These countries are Finland, Sweden, Scotland, England, Italy, and Catalonia and in Table 1 below, a summary of some health resources indicators is given. Sweden is the country that invests more in health and has the highest ratio in nurses and physicians. Finland is the one with the highest ratio in terms of hospital beds. The majority has a shared implementation model, but in Italy and Catalonia regions are fully responsible. Sweden, Finland, and Catalonia have a similar PHC organizational model, whereas in Italy, Scotland, and England PHC is organized as independent contractors. According to the ODHIN assessment report (22), the integration of the management of HHAC in the PHC system (scale 0–10) is best in Sweden, followed by Catalonia/Spain, and in secondary health care it is best in Catalonia/Spain, followed by England/UK.

**Table 1:** **Health system key characteristics**.

	Finland	Sweden	UK	Italy	Spain
Population^a^	5,413,971	9,519,374	63,705,000	59,539,720	46,146,580
Total expenditure on health/capita, US$ purchasing power parity, 2011^a^	2,544.7	3,203.6	2,821.1	2,344.5	2,244.2
Health resources density per 1000 population (head counts)^a^	10.45 (Nurses)	11.09 (Nurses)	8.21 (Nurses)	(Nurses)	5.24 (Nurses)
	2.72 (Physicians) – 2008	3.92 (Physicians)	2.75 (Physicians)	3.85 (Physicians)	3.82 (Physicians)
	5.3 (Hospital beds)	2.62 (Hospital beds)	2.81 (Hospital beds)	3.4 (Hospital beds)	2.97 (Hospital beds)
Type	Compulsory tax-based	Compulsory tax-based	National taxation	General taxation	Tax-based
Planning/implementation	National planning, local (municipalities) implementation	Central state, regions and local health authorities (shared responsibility)	Country (England, NI, Scotland, and Wales) deliver services through public providers	Central state, regions, and local health authorities (shared responsibility)	Central state defines minimum requirements and coordinates, autonomous communities are fully responsible
Health care provision	PHC centers are multidisciplinary and public owned and provide (primary care, preventive care and public health services)	PHC services deliver both basic curative care and preventive services through local health centers	PHC is provided by GPs in group practices (three per practice)	GPs and pediatricians working as independent contractors provide primary health care	PHC centers are multidisciplinary and public owned and provide primary and preventive care
Self-declared unmet needs for medical examination (EU rate = 3.4%)^b^	Above	Below	Below	Above	Below
Integration of the management of hazardous and harmful alcohol consumption in the primary and secondary health care system (scale 0–10)^c^	5/5	10/4	5/6 (England only)	5/4	8/8

^a^OECD Health Statistics, 2013 – http://stats.oecd.org/index.aspx?DataSetCode=SHA

*^b^Eurostat statistics on income and living conditions, 2012*.

^c^ODHIN assessment tool – report, 2013 – http://www.odhinproject.eu/project-structure/wp6.html

During the last decade all of these countries undertook major reforms of their healthcare systems in the five key identified areas: strengthening health care financing, continuum of care, quality of HSs, linkage with community, and advances in public health. This process has slowed down or even stopped in Catalonia and Italy due to the recession and the cuts in the HSys.

## Case Studies Analysis: What has been Done

Interest in the SBI in the six countries started early, especially in Sweden and Scotland, where the first studies began in the early 80s. All the countries, except Sweden and Scotland, took part in the WHO Collaborative Study (Table 2). Countries joined in different phases, England in Phase II (SBI trial), Italy and Catalonia in Phase III (best ways to achieve wide implementation), and Finland in Phase IV (country-wide SBI implementation strategies).

**Table 2:** **SBI programs characteristics**.

	Finland^a^	Sweden	Scotland^b^	England	Italy	Catalonia/Spain
Origin	Late 90s. Phase IV of the WHO Collaborative Project^c^. PHC and occupational health	Early 80s Malmö study. Risky drinking Project (2004–2010) in PHC, maternity and occupational health care	Early 80s DRAM Study. Scotland performance management target (H4:Heat target)^d^	Late 80s. Phase II of the WHO collaborative project. SIPS trials (PHC, emergency departments and criminal justice settings	Early 90s. Phase III strand I of the WHO Collaborative Project	Mid 90s. Phase III-strand III of the WHO Collaborative Project. Phase IV on implementation started in 2002
National guidelines	Yes. Part of other clinical care guidelines	Yes. Stand alone guidelines (GP)	Yes. Stand alone guidelines (GP and nurses). The management of harmful drinking and alcohol dependence in primary care^f^	Yes. Stand alone guidelines (GP and nurses) NICE guidance on the prevention of hazardous and harmful drinking plus a Nationally Directed Enhanced service	Yes. Stand alone guidelines (GP). PHEPA^e^ adapted at national level	Yes. Stand alone guidelines (GP and nurses). PHEPA^e^ adapted at national level and PAPPS^f^
Professionals	Both GP (1,000) and nurses (2,000)	Both GP, residents in family medicine and district nurses	GP and other PHC professionals (practice and community nurses and health visitors)	Both GP and nurses	GPs, psychiatrists, family advice bureau from PHC; psychologists, professional from the Ser.T.S. and workplace	Both GP and nurses
Screening	Opportunistic screening with AUDIT	AUDIT	Clinical presentations and new registrations. Abbreviated forms of AUDIT (e.g., FAST), or CAGE plus two consumption questions, should be used in primary care when alcohol is a possible contributory factor	Targeted screening with AUDIT and AUDIT-C	Targeted screening with AUDIT and AUDIT-C on a voluntary basis	Universal with existing tools (quantity and frequency) in medical records and AUDIT (voluntary)
Brief intervention	FRAMES adapted BI	Feedback and BI. MI-principles	FRAMES adapted BI (10 min)	Simple structured advice and brief behavioral counseling	Based on PHEPA guidelines (FRAMES adapted BI)	FRAMES adapted BI
Training^g^	Both vocational and continuing medical education (GP and nurses)	Only vocational training (GP). During the project: half and whole day information seminars and network meetings	Training of trainers (100). NHS health Scotland trained over 3200 practitioners (Training manual, DVD and a national competency	Partially available vocational training and continuing medical education (GP and nurses). During the project: training of trainers (How much is too much package)	Only vocational training (GP). During the project: training of trainers (PHEPA training manual) and continuing medical education (ECM)	Both vocational and continuing medical education (GP and nurses) Training by peers in the PHC (Beveu Menys package)
Incentives or part of normal salary^g^	Part of normal salary	Incentives	Incentives	Part of normal salary	Part of normal salary	Small incentives
Support for managing SDA in specialized treatment facilities	Yes	Yes	Yes. Access to relapse prevention treatments	Yes. Evidence-based care pathway for different levels of alcohol-related risk harm and dependence	Yes	Yes. Strategy on coordination between PHC and specialist services for alcohol dependence
Monitoring and evaluation	Pre-post. Mailed questionnaire to all PHC physicians (2002–2007). Face-to-face interviews (2008) (self-report measures). 25% of Finnish population but concerted attempt to cover the whole country	Pre-post. Telephone-administered questionnaire to general population (2006–2009) (self-report measures)	Trials, case studies to assess extend of adoption and reach	National audit office report. annual care quality commission report	Not on SBI implementation but on alcohol consumption, mortality, attributable hospital discharges and on public specialist alcohol service activities (125/2001 law on alcohol)	Annual screening rates (contract with PHC providers)
Governmental funding for services for HHAC	Yes	Yes	Yes	No	Yes	Yes
Specific national policy	Yes. Finnish alcohol program (2004–2007)	Government initiative	Health service target of delivering 149,449 BI 2008/2009–2010/2011	National alcohol strategies (2 since 2004)	Frame law on alcohol 125/2001 National alcohol and health plan (PNAS) National prevention plan (PNP) National health plan (PSN)	No but included in the health Plan (2012–2016) and in the drug prevention plan
Presence of country coalition for the management of HHAC	Yes	Yes. Cooperation with 21 county councils. Supervision by the professional organizations, local authorities, Hospitals, etc	–	Yes	Yes. National Observatory on Alcohol – CNESPS, Istituto Superiore di Sanità (with funding from the MoH and the Presidency of the Council of the Ministries, Dept of antidrugs policies)	Yes. Program on Substance Abuse of the Department of Health (full time nurse and half time administration staff) in collaboration with PHC providers and Catalan Society of Family and Community Physicians and Nurses
General and family practice availability and accessibility Mean = 6^g^	Mean	Mean	–	Below	Below	Above
Professionals accountability GP Mean = 5.4 Nurses Mean = 4.5^g^	Above/above	Below/below	–	Below/above	Below/below	Above/above

^a^WHO-Phase IV, Finland report – http://www.gencat.cat/salut/phaseiv/finland.htm

^b^Alcohol Brief Interventions: communication and Guidance – http://www.healthscotland.com/topics/health/alcohol/alcohol-brief-interventions-communications-and-guidance.aspx

^c^WHO-Phase IV website: http://www.gencat.cat/salut/phaseiv/index.htm

^d^SIGN no. 74 (2003) – http://www.sign.ac.uk/guidelines/fulltext/74/index.html

^e^PHEPA guidelines: http://www.phepa.net/units/phepa/html/en/dir361/doc13210.html

^f^Programa de actividades preventivas y de promoción de la salud (PAPPS) – http://www.papps.org/upload/file/Grupo_Expertos_PAPPS_2_2.pdf

^g^ODHIN assessment tool – report – A description of the available services for the management of hazardous and harmful alcohol consumption (2013) – http://www.odhinproject.eu/project-structure/wp6.html

Phase IV began in 1999 and ended in 2006. While participating countries shared the same objective the specific design and procedures varied among participating countries in order to take account of different country specific needs, factors, and policies and PHC organizational models (23). In Table 2 below, you can find the main characteristics of the implementation that has taken place.

The so-called treatment gap, the proportion of people who actually access treatment out of those who need it, has been reported in the majority of the countries as one of the main motivations to implement SBI. In the study from Wolstenholme et al. (24) across six European countries studied, there was a great variation in the HSys and treatment provision for alcohol use disorders, with the proportion of people in need of treatment who actually access it ranging from 1 in 25 to 1 in 7. Italy was the country with highest access to treatment (23.3%) and England (7.1%) had one of the lowest. Interestingly, in Sweden the SBI project was launched against a backdrop of increasing alcohol consumption since the country’s entry in the EU in 1995 (15). In Scotland, a substantial rise in alcohol-related harm is reported too (25).

As detailed in Table 2, SBI programs share some communalities (AUDIT as screening tool and FRAMES adapted brief intervention), especially among those that participated in the WHO Collaborative project, but its implementation has been adapted to the country HSys organization (PHC settings structure, professionals involved, referral pathways). An important issue is that regardless of the origins, governments have been involved in the SBI program implementation mainly by endorsing national guidelines or policies and providing specific funding for HHAC. As far as we know, only Scotland established a national target and incentivized accordingly. It is not clear, however, if sustainability actions are undertaken in order to maintain results obtained in the different countries.

Italy and Catalonia have based their evaluation more on continuous monitoring strategies than on specific research trials or studies; UK and other countries have followed a much more formal monitoring including a national Audit. Studies on fidelity to national guidelines in such countries do not exist.

Taking into consideration the main conclusions of the Odhin assessment exercise (22), success in the wide implementation of SBI depends on a number of factors: the presence of a formal partnership or coalition to support the process, the integration of the management of the SBI in the health care system, the provision of a formal, mandatory on-going training and medical education on SBI, the existence of written alcohol policies funded SBI research projects (cost-effectiveness, fidelity, quality of advice, evaluation surveys, performance records, etc.), available guidelines and protocols provision of materials and incentive measures, support by specialists services, etc. Furthermore, it is essential that specific activities should be devoted to the dissemination of available sources of knowledge, research results, and information to health care providers together with the provision of materials and tools as well as incentive measures aimed at ensuring that prevention, particularly SBI, is implemented in PHC and supported by specialist services according to a real networking of the available servicers and competencies.

## Proposal for the Future: The WHO-Euro Strategy on HSys for BI

Alcohol, in contrast to other behaviors and lifestyles poses important challenges to the HSys, mainly because of moral prejudices existing in our society, to the fact that alcohol consumption is culturally and socially determined and to the fact that there in some cases it is associated to brain malfunctioning or a brain disease. This together with the barriers in every day practice to sustaining commitment such of lack of time, lack of training and resources, a belief that patients will not take advice to change drinking behavior and a fear of offending patients by discussing alcohol (26, 27) has resulted in HSys oriented toward an individualized, passive, and an illness-centered model of health care in which SBI implementation is utopic.

Coming to this point, it is clear that unless the HSys adopts more holistic and patient-centered implementation models, the SBI implementation on HHAC will not be achieved and sustained despite all the research and efforts done. In this direction, we would like to emphasize the relevance of the contributions made by:

### Tblisi resolution

Behavior Change strategies and health: the role of HSys (6) that acknowledges the fact that behavior-related risk factors have become the leading causes of morbidity and mortality and that they cannot be seen in isolation, as they mostly are inextricably connected with the social determinants of health.

### Tallinn charter

Health system for health and wealth (6) that stresses that effective primary care is essential to provide a platform for the interface of HSs with communities and families and for intersectoral cooperation and health promotion that HSys should integrate targeted disease-specific programs into existing structures and services and that HSys need to ensure a holistic approach to services, involving health promotion, disease prevention, and integrated disease management programs, as well as coordination among a variety of providers, institutions, and settings, irrespective of whether these are in the private or public sector and including primary care, acute, and extended care facilities and people’s homes, among others.

Thus, talking specifically about the management of HHAC, the Tblisi resolution tells us that complex factors influencing alcohol behavior change should be taken into account in order to design proper interventions (see Table 3).

**Table 3:** **Common factors influencing behavior change and their implications for intervention design [adapted from WHO European Ministerial Conference on Health Systems (28)]**.

Factors	Design implication
A desire for change must be present in the audience	There is a need both to create a demand for positive change and to create the conditions to enable people to make positive choices
Participatory involvement leads to greater behavioral change effects	Interactive engagement strategies and the development of coalition approaches to change should be part of all behavior change interventions
People are often motivated to do the “right thing” for the community as well as for themselves and their families	Programs should encourage and incentivize socially responsible behavior and penalize behaviors that are not socially responsible
Social relationships, social support, and social norms have a strong and persistent influence on behavior	Incorporating peer and family support strategies into individual risk change programs increases likely success
Change is usually a process not an event	Programs should be sustained over time and tailored to the needs of different groups
Psychological factors, beliefs, and values influence how people behave	Programs need to address values and beliefs, as well as information and knowledge acquisition
People can be “locked into” patterns of behavior and need practical help to break them	Policy and services need to be designed to meet the specific needs of different communities, in order to help them change engrained habits
Change is more likely if an undesired behavior is not part of an individual’s life situation coping strategy	Create incentives, offer practical support for change, and give positive reinforcement. Provide alternative forms of support and reinforcement to aid behavior change
People’s behavior is influenced by their physical and social environments	There is a limit to a person’s capacity to change, if the environment militates against the desired change; conditions and incentives for change must therefore be created, in addition to giving messages and advice and building personal skills
People’s perception of their vulnerability to a risk and of its severity is key to understanding behavior	There is a need to develop individual and community understanding of risk and vulnerability in relation to major threats
Perceptions of the effectiveness of the recommended behavior change are key factors affecting decisions to act	Programs should seek to ensure that people understand the scale of the rewards associated with positive behavior change
The more beneficial or rewarding an experience, the more likely it is to be repeated	Reinforcing and incentivizing positive behavior in the short term should be part of any change program
People are loss-averse: they will put more effort into retaining what they have than into acquiring new assets	Programs should emphasize the advantages of positive behaviors that enable a continuation of immediate benefits, rather than long-term gains
People often rely on mental short cuts and trial-and-error to make decisions, rather than on rational computation	Programs should develop a deep understanding about what will motivate people to change and how they perceive specific issues

All the factors listed above are applicable to alcohol behavior change and to the design of alcohol interventions. The behavioral change model acknowledges the important role that, for example, the physical and social environments, the social relationships, and the social norms play on the alcohol consumption and as a result of this, alerts on the limit to a person’s capacity to change, if the environment militates against the desired change; and the importance to create conditions and incentives for change, in addition to giving messages and advice and building personal skills. This model also stresses that some people are just physiologically incapable of drinking moderately and that in such cases actions to empower (29), to increase self-esteem (30) and resilience (31) of the harmful drinkers should also be implemented to increase effectiveness. Thus, behavior change could benefit from information, education, and capacity building interventions, at community and, especially, at individual level.

In addition to that, according to the Tallinn charter, it is clear that the implementation of SBI in PHC alone would not produce the effect we are aiming for.

From the model proposed (see Table 4), it is clear that in order to introduce such individually oriented strategies by PHC it is essential to embed them into settings and systems oriented strategies such as health promotion approaches and community and population strategies such as mass media campaigns regulation and legislation and capacity building.

**Table 4:** **Components of a comprehensive approach to health behavior change [adapted from WHO European Ministerial Conference on Health Systems (28)]**.

**Community and whole of population strategies**			
Legislation and regulation	Environmental Change (footpaths, cycleways, lighting)		Mass media campaigns
Community partnerships	Community capacity building	Existing community structures and leadership	Culturally and behaviorally tailored programs
**Settings and systems oriented strategies**			
Setting intervention: workplaces	Setting intervention: educational institutions	Setting intervention: primary health care	Setting intervention: home and family
Social support, e.g., walking group	Telephone counseling	Signs/cues at points of decision-making	Internet
**Individually oriented strategies**			
Personal goal-setting	Self-monitoring, e.g., daily–diary	1:1 or group counseling	Brief advice from GP or health professional

Taking all this into account, the following considerations could be made.

- From a Public Health point of view, to increase the effectiveness of any alcohol risk reduction all these aspects need to be taken into consideration and the respective stakeholders need to be involved in a wide, holistic, intersectoral approach. Social and HSs, culture and education, pharma industry, local authorities, private sector, general population representatives, and the economic sector are only some of the participants that need to be involved.
- From an individual point of view, primary healthcare and general practitioners (GPs), in particular, need to participate because they can take care of all those issues and work for behavioral change in an effective way (32, 33). They provide lifelong, continuing, co-ordinated, and community oriented care to their patients and are widely seen by them as their most trusted health providers. They are also recognized as being the gatekeepers in many European HSys and they are the only health professionals that have the formal role and possibility to recover information on every health determinant, educate, and provide support to their patients. Genetics, mental health, family situation, culture, religious beliefs, and socio-economic positions can be easily accessed by these experts assuring thus a holistic approach. In their everyday work, GPs should know that increasing awareness and knowledge is essential for behavioral change but they are seldom sufficient to promote a sustainable modification in health behaviors. The ability to change is also influenced by each citizen’s values, attitudes and norms, self-perception and capacity for sustaining the change, expectations of success and failure before embarking on a change program.
- Apart from increasing health literacy and managing health issues, we need to influence individual attitudes and the level of confidence, which are more bound to health determinants such as culture, social models, economic, and working conditions. Individual health needs should be addressed and also individual resources, in a non-medical, positive, health promotion approach.

## Conclusion

This review contextualizes the importance of the implementation of SBI in the context of effective alcohol policies, summarizes the main effectiveness and cost-effectiveness evidence, and describes the major accomplishments achieved with nation-wide SBI implementation programs in Europe. This review also provides the means to think of different approaches if more effective AUDs strategies are to be proposed at European level. Social, economic, and health promotion points of view are also presented as important aspects to be explored for the good outcome of any AUDs strategy.

Major and diverse issues were identified in this review:

- Implementation is still not Country- and Europe-wide. Pilot experiences should be generalized. The recent trial results strongly reinforce the already expressed suspicion that it is extremely difficult to get health professionals to deliver SBI; The ODHIN assessment tool shows that, in 2012, EIBI was still not the norm in daily consultation in PHC and that more resources are needed to overcome the main obstacles.
- Enduring behavior change and improvements on biochemical and biometric measures are unlikely after a single routine consultation with a clinician trained in behavior change counseling, without additional intervention.
- A tailored, implementation multi-faceted program aimed at improving general practitioner management of alcohol consumption showed little evidence to support the use of such an intensive implementation program to improve the management of harmful and hazardous alcohol consumption in primary care.
- Despite the efforts made toward the country-wide implementation of SBI programs, comparisons are difficult, not only due to the different context and implementation strategies used but also by the diversity of the outcome and output indicators used. Little can be said about what works, what does not and in what contexts.
- As stated by many authors (11, 34), further evaluation of all the programs under a common evaluation framework like the reach, effectiveness, adoption, implementation, and maintenance (RE-AIM) or the CFIR that go beyond the standard models of technology diffusion would be essential in order to extract more structured ideas on the implementation needs. Authors (8, 35) suggest that new modes of delivery such as via internet may help to surmount some of the challenges of wide dissemination, such as strains on expertise, time, and resources but still more research has to be done on its efficacy and effectiveness. In addition to that, in order to achieve a population-wide dissemination it is essential to involve other health and social settings and actors, thus, expanding SBI evidence is essential. In this sense, initiatives such as the “BISTAIRS project” – will provide useful information on how to foster the implementation of SBI for AUDs in a variety of settings (PHC, workplace HSs, emergency care, and social services) and extending best practices in Europe (36, 37).
- Implementing SBI through a PHC system approach is important because addressing risky drinking is a complex issue, involving different actors from different parts of the society. Families, local communities, and work environments are the usual settings where those risks are generated and PHC is the right place to understand the conditions that bring people to adopt unhealthy lifestyles.

Taking into account all these elements listed above, leads inevitable to the need to reframe SBI. The challenge is how to do it without impacting on its cost-effectiveness and practicability in PHC to reduce alcohol health risks (20, 38). Some suggestions will be:

- To broaden it to a brief motivational intervention, which could allow professionals to understand and evaluate individual health determinants and self-esteem and to determine people’s motivations to change by addressing patient’s importance and confidence to change and help them to understand the individual conditions underlying their risky drinking.
- To strengthen the links with territorial services as an essential way to provide structural support, when needed.
- To broaden brief interventions to allow a more traditional, pharmacological treatment, more in line with professional’s (especially GPs) attitudes and views.
- To abandon simplistic and potentially unhelpful positions of putting on each individual patient the sole responsibility and decision to adopt healthier behaviors to avoid ill health.
- To integrate peer and family support strategies into individual risk modification in order to increase the SBI success (39).
- To propose alcohol SBI within the broader issues of all lifestyles and within the context of a global cardiovascular and cancer risk reduction. Asking about alcohol drinking, food intake or tobacco smoking, just like asking about blood pressure, can be an easy step forward to increase effectiveness.
- To integrate brief interventions with on-going practical support for structural changes performed by other actors (social services, community networks, psychologists, psychiatrists, etc.) could ease the work of primary healthcare and allow a better management of AUDs.
- To take advantage of new information and communications technologies (ICT) to help addressing the problem and enabling patients and health care providers to work as co-producers of health. Without abandoning completely the traditional face-to-face engagement, there is mounting evidence of the effectiveness of delivering aspects of healthcare using the Internet and mobile phone applications for the promotion of healthier lifestyles (smoking cessation, healthier drinking choices, and weight loss) (40, 41). Work is also underway on the development of digital technologies to enable patients with long-term conditions such as AUDs, obesity, and chronic obstructive airways disease to engage more actively in the management of their own health and trials are being undertaken to evaluate the potential of these applications to deliver benefits in relation to patient satisfaction and wellbeing as well as clinical outcomes (21).
- To work closely in connection with patients and the public as well as different stakeholders (medical and social, the pharmaceutical industry, public health authorities, ICT and m-health actors, health economists, health insurers) to understand people’s attitudes and motivations, as well as barriers to change, perceived or real, in a real community holistic approach, to address health determinants and explore new, co-produced health models. Be involved in alcohol risk management considering the need to reduce stigma by including alcohol in usual care, with other lifestyle related risks and in the broader question of cardiovascular risk management.
- To create more appealing specialist services to help reducing stigma associated to AUD.
- The fact that it is difficult to effectively implement and maintain SBI strategies should bring policy makers to explore new possibilities, linking different stakeholders with different approaches, and trying new methodologies, including the provision of appropriate training, incentives, and implementation strategies.

In summary, alcohol use is a complex issue, at least as much so as hypertension or diabetes. Thus, thinking that a single intervention, even if effective, such as SBI, could solve the problem is, to our point of view, naïve, and restrictive. Future strategies should aim at broadening the perspective from an individual and a HSys point of view. HSs are important in addressing AUDs but only if individual tailored strategies are proposed, taking into consideration all the complexity of human being and his environment in a Health System approach (42).

## Conflict of Interest Statement

The authors declare that the research was conducted in the absence of any commercial or financial relationships that could be construed as a potential conflict of interest.

## Footnotes

^1^Health promotion is the process of enabling people to increase control over, and to improve, their health. It moves beyond a focus on individual behavior toward a wide range of social and environmental interventions (WHO definition).

^2^A health system is the sum total of all the organizations, institutions, and resources whose primary purpose is to improve health.
